# Design of a new multi-epitope vaccine against *Brucella* based on T and B cell epitopes using bioinformatics methods

**DOI:** 10.1017/S0950268821001229

**Published:** 2021-05-25

**Authors:** Zhiqiang Chen, Yuejie Zhu, Tong Sha, Zhiwei Li, Yujiao Li, Fengbo Zhang, Jianbing Ding

**Affiliations:** 1 Department of Immunology, College of Basic Medicine, Xinjiang Medical University, Urumqi, 830011 Xinjiang, China; 2 Department of Reproductive Assistance, Center for Reproductive Medicine, The First Affiliated Hospital of Xinjiang Medical University, No. 393, Xinyi Road, Urumqi, 830011 Xinjiang, China; 3 Clinical Laboratory Center, Xinjiang Uygur Autonomous Region People's Hospital, Urumqi, 830001 Xinjiang, China; 4 Department of Clinical Laboratory, The First Affiliated Hospital of Xinjiang Medical University, No. 393, Xinyi Road, Urumqi, 830011 Xinjiang, China; 5 State Key Laboratory of Pathogenesis, Prevention, Treatment of Central Asian High Incidence Diseases, the First Affiliated Hospital of Xinjiang Medical University, No. 393, Xinyi Road, Urumqi, 830011 Xinjiang, China

**Keywords:** *Brucella*, multi-epitope vaccine, Omp22, Omp19, Omp28, Omp22, outer membrane protein, Omp19, outer membrane protein 19, Omp28, outer membrane protein, NCBI, National Center for Biotechnology Information, DNA, desoxyribonucleic acid, LPS, lipopolysaccharide, Cu/Zn SOD, Cu-Zn superoxide dismutase, TCR, T cell receptor, BCR, B cell receptor, C-score, confident score, HBHA, heparin-binding haemagglutinin, TM, template modelling, RMSD, root mean square deviation, MHC, major histocompatibility complex, GRAVY, grand average of hydropathicity, 3D, three-dimensional, HLA, human leucocyte antigen, CTLs, cytotoxic T lymphocytes, ACC, auto cross covariance, *E. coli*, *Escherichia coli*

## Abstract

Brucellosis is one of the most serious and widespread zoonotic diseases, which seriously threatens human health and the national economy. This study was based on the T/B dominant epitopes of *Brucella* outer membrane protein 22 (Omp22), outer membrane protein 19 (Omp19) and outer membrane protein 28 (Omp28), with bioinformatics methods to design a safe and effective multi-epitope vaccine. The amino acid sequences of the proteins were found in the National Center for Biotechnology Information (NCBI) database, and the signal peptides were predicted by the SignaIP-5.0 server. The surface accessibility and hydrophilic regions of proteins were analysed with the ProtScale software and the tertiary structure model of the proteins predicted by I-TASSER software and labelled with the UCSF Chimera software. The software COBEpro, SVMTriP and BepiPred were used to predict B cell epitopes of the proteins. SYFPEITHI, RANKpep and IEDB were employed to predict T cell epitopes of the proteins. The T/B dominant epitopes of three proteins were combined with HEYGAALEREAG and GGGS linkers, and carriers sequences linked to the N- and C-terminus of the vaccine construct with the help of EAAAK linkers. Finally, the tertiary structure and physical and chemical properties of the multi-epitope vaccine construct were analysed. The allergenicity, antigenicity and solubility of the multi-epitope vaccine construct were 7.37–11.30, 0.788 and 0.866, respectively. The Ramachandran diagram of the mock vaccine construct showed 96.0% residues within the favoured and allowed range. Collectively, our results showed that this multi-epitope vaccine construct has a high-quality structure and suitable characteristics, which may provide a theoretical basis for future laboratory experiments.

## Introduction

*Brucella* is a Gram-negative intracellular pathogen that causes brucellosis [1], it usually can be divided into 12 species in nature, including six so-called classic *Brucella* species, namely *B. melitensis, B. abortus, B. suis, B. canis, B. ovis* and *B. neotomae*, and six newly discovered *Brucella* species from wild mammals, amphibians and fish, namely *B. microti, B. pinnipidialis, B. ceti, B. inopinata, B. papionis* and *B. vulpis.* In the genus *Brucella, B. melitensis, B. abortus* and *B. suis* have good clinical significance [2–4]. Brucellosis in animals manifests itself in miscarriages and reduced fertility and is transmitted to humans by inhaling aerosolised bacteria or ingesting contaminated derivatives. Clinical symptoms of human brucellosis include undulant fever, arthritis and general weakness [5, 6]. At the present medical level, it is difficult to completely eliminate *Brucella* [7]. Therefore, the vaccine is an ideal way to prevent *Brucella* infection [8]. Currently, there are no *Brucella* vaccines for humans, and the live-attenuated vaccines designed for animals have many defects, including interference with serological testing and human infectivity [9]. Therefore, the subunit vaccine with no hidden danger and good protective effect has become a new hotspot in brucellosis research. The research of *Brucella* subunit vaccine mainly includes desoxyribonucleic acid (DNA) vaccines, lipopolysaccharide (LPS) vaccines and protein vaccines [10, 11]. With the rapid development of bioinformatics technology, epitopes of different antigens can be constructed as a novel vaccine with good immune effects.

In previous studies, a series of different proteins from *Brucella* has been used to identify immunodominant antigens against *Brucella* infection, including outer membrane proteins [12], flagellar proteins [13–15], L7/L12 ribosomal proteins [16] and Cu−Zn superoxide dismutase (Cu/Zn SOD) [17], etc. The Omp22 protein is an immunodominant antigen, belonging to the Omp25/Omp31 family of proteins. It is highly conserved among various species of *Brucella* and is related to the infectivity of *Brucella*. Studies have shown that the Omp22 protein is similar to the LPS of *Brucella* and induces an immune response in the body [18]. The Omp19 is exposed at the cell surface of *Brucella* spp, and it can be employed for protection against *Brucella* [19]. The Omp28 is also an important outer membrane protein of *Brucella*. It is highly conserved among various genera. It has been reported that the Omp28 peptide with CpG oligonucleotide as an adjuvant can induce an immune response mediated by IgG2a type, indicating that the Omp28 can induce the body to produce large amounts of IgG antibodies [20]. It has been known that vaccines constructed from a single protein stimulate poor immune responses and that multiple protein combinations enhance the vaccine's immune response [21]. Therefore, in this study, based on the T/B epitopes of Omp22, Omp19 and Omp28, a multi-epitope vaccine against *Brucella* was constructed. To verify the availability of the vaccine construct, the tertiary structure, secondary structure, physical and chemical properties, solubility, antigenicity and allergenicity of the vaccine construct were analysed by various bioinformatics software. The results indicated that the multi-epitope vaccine construct could be used as a candidate protein against *Brucella.*

## Methods

### Amino acid sequence of the protein

The amino acid sequences of the Omp22, Omp19, Omp28 were searched in the GenBank database (https://www.ncbi.nlm.nih.gov/genbank/).

### Prediction of signal peptide

SignalP-5.0 Server [22] was used to predict the signal peptide of the protein sequence. SP (Sec/SPI) is related to the type of signal peptide predicted; CS represents the cleavage site; Other: the probability that the sequence does not have any kind of signal peptide.

### Identification of hydrophilic residues and surface accessible

Immunoglobulins usually bind to the water-accessible regions of antigens. Thus, the predicted epitopes should ideally be located in the highly hydrophilic region with many accessible residues. Surface accessible and hydrophilic regions of the protein were determined using the ProtScale software [23] and marked these areas through UCSF Chimera software [24].

### Prediction of tertiary structure

The I-TASSER server [25] automatically generated high-quality tertiary structure models of protein molecules from amino acid sequences. The structure and function of proteins were predicted by I-TASSER based on analytic hierarchy process. Here, we used the confidence score (C score) to evaluate the predictive model quality. The C score is in the range of – 5 to 2, where the higher C score, the higher credibility of the model. The template modelling (TM) score was used to deal with some error-sensitive root mean-square deviation (RMSD) problems. A TM score <0.17 indicates random similarity, and only a TM score>0.5 could indicate a correct topology model. These cutoff values did not depend on the length of the protein.

### B cell epitope prediction

To ensure the accuracy of the prediction of B cell epitopes, we used various prediction software, including COBEpro (http://scratch.proteomics.ics.uci.edu/), SVMTriP (http://sysbio.unl.edu/SVMTriP/prediction.php) and BepiPred (http://www.cbs.dtu.dk/services/BepiPred-1.0/). The overlapping sequences of the top 10 from at least two software were chosen as B cell dominant epitopes.

### T cell epitope prediction

T cells can be divided into CD4^+^ T cells and CD8^+^ T cells, which are restricted by the major histocompatibility complex (MHC) in identifying epitopes. Human MHC is called the human leucocyte antigen (HLA) gene complex. CD4^+^ T cells recognise antigenic epitopes consisting of 9–22 amino acid residues, which are limited by their HLA-II molecules and differentiate into T helper cells after activation. CD8^+^ T cells recognise epitopes consisting of 8–12 amino acid residues, which are limited by HLA-I molecules, and differentiate into cytotoxic T lymphocytes (CTLs) after activation. Therefore, it was necessary to predict CD4^+^ and CD8^+^ T cell epitopes, respectively, when predicting T cell epitopes. We selected HLA-A * 0201 and HLA-A * 2402 discerned by HLA-I and HLA-DRB * 0701 and HLA-DRB * 0901 discerned by HLA-II, which were the four most common alleles in North China. Some different online software was used to predict T cell epitopes, including IEDB (http://www.iedb.org/home_v3.php), SYFPEITHI (http://www.syfpeithi.de/bin/mhcserver.dll/epitopeprediction) and RANKPEP (http://imed.med.ucm.es/Tools/rankpep.html). We listed the top 10 high-score epitopes for each prediction software and selected three software overlapping sequences as T cell dominant epitopes for the proteins.

### Predicting immunogenicity and antigenicity of CD8^+^ T cell epitopes

Epitope/HLA complexes should be capable of eliciting strong immune responses. Therefore, we used the HLA I immunogenicity prediction tool of the IEDB server, the parameters were set to default. The antigenic properties of all CD8^+^ T cell epitopes were analysed using VaxiJen 2.0 Server [26] with a threshold of 0.5. Finally, we selected CD8^+^ T cell epitopes with immunogenicity and antigenicity for the next step (http://tools.immuneepitope.org/immunogenicity/).

### Multi-epitope vaccine sequence construction

The predicted T/B dominant antigen epitopes were linked by some amino acid linkers. To enhance the immunogenicity of the epitope vaccine, heparin-binding haemagglutinin (HBHA) conservative sequences were added to epitope sequences as the carrier [27]. It is also known that the PADRE peptides can induce CD4^+^ T cells and enhance the immune function of the vaccine construct [28].

### Assessment of allergenicity, antigenicity and solubility

The allergenicity of the vaccine construct was predicted by SDAP [29]. SDAP can predict the cross-reactivity of candidate vaccines and known allergens through the allergenicity rules of WHO. We chose the default parameter, which was a similarity of more than 35% for full-length sequences, and an E cutoff of 0.01 when the sliding window was aligned to 80 amino acids, and 6 adjacent short amino acid sequences matched with known allergens. The antigenicity of the vaccine construct by VaxiJen 2.0 server. This server relies on auto cross covariance (ACC) transformation, and alignment-independent predicted antigenic epitopes by physiochemical properties of proteins. The SOLpro [30] was used to predict the solubility of the vaccine construct. Based on the multiple representation of the first-order amino acid sequence，two-stage SVM architecture was adopted. The final result is to summarise the prediction with 74% overall accuracy at the corresponding probability (³0.5).

### The secondary structure prediction

The proportions and distributions of *Alpha helix*, *Beta turn, Random coil, Extended strand* in vaccine construct sequence were predicted and analysed using SOPMA online analysis software [31].

### Prediction of various physicochemical properties

The online tool ProtPararm from Expasy (http://www.expasy.org/protparam/) was used to analyse the physicochemical properties of the vaccine, including theoretical isoelectric points, molecular weight, hydrophilicity, atomic composition and extinction coefficient. The physical and chemical properties from the pk values of amino acids were calculated by ProtPararm software.

### Construction of the tertiary structure of the vaccine construct

The I-TASSER online software was used to construct the vaccine's tertiary structure, which was validated by Ramachandran diagrams in the RAMPAGE webserver. (http://mordred.bioc.cam.ac.uk/~rapper/rampage.php). The Ramachandran plot is a method to show the allowed and disallowed dihedral angles *psi (ψ)* and *phi (ϕ)* of amino acid. It is calculated according to van der Waal radius of the side chain.

## Results

### Amino acid sequence of protein

Obtaining the Omp22 protein sequence from GenBank (Accession: AAS84601.1): MFKRSITAAALGAAVMAFAGSAFAADMMGGTDYTYNDPVAAGPHDWSGNYVGAQVGGSSSKFPSPFASRTGALGGIVVGKNMQNGNIVFGAELEGNFAEAEHRIGHGGTLQQSWNGNAKGKVGYTFDKTLVYGTAGYGVTRFKAKDNTTSASGWEGGVLIGAGVEQALSGPLSVKAEYDFQRFNDVKSQVNGIEQRNNLKNHSIKAGLNYKF Obtaining the Omp19 protein sequence from GenBank (Accession: ERU25360.1): MGISKASLLSLAAAGIVLAGCQSSRLGNLDNVSPPPPPAPVNAVPAGTVQKGNLDSPTQFPNAPSTDMSAQSGTQVASLPPASAPDLTPGAVAGVWNASLGGQSCKIATPQTKYGQGYRAGPLRCPGELANLASWAVNGKQLVLYDANGGTVASLYSSGQGRFDGQTTGGQAVTLSR Obtaining the Omp28 protein sequence from GenBank (Accession: AEF59021.1): MNTRASNFLAASFSTIMLVGAFSLPAFAQENQMTTQPARIAVTGEGMMTASPDMAILNLSVLRQAKTAREAMTANNEAMTKVLDAMKKAGIEDRDLQTGGINIQPIYVYPDDKNNLKEPTITGYSVSTSLTVRVRELANVGKILDESVTLGVNQGGDLNLVNDNPSAVINEARKRAVANAIAKAKTLADAAGVGLGRVVEISELSRPPMPMPIARGQFRTMLAAAPDNSVPIAAGENSYNVSVNVVFEIK

### Signal peptide of proteins

**Fig. 1.**
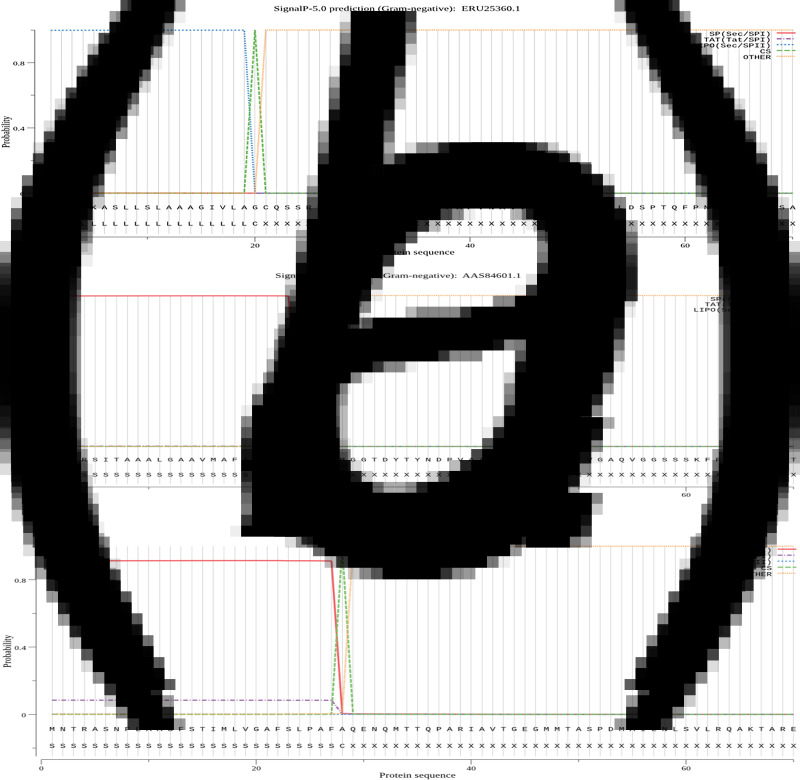
Signal peptide of proteins using SignalP-5.0 analysis. SP (Sec/SPI): type of signal peptide predicted; CS: the cleavage site; Other: the probability that the sequence does not have any kind of signal peptide. (a) The signal peptide prediction of Omp19: MGISKASLLSLAAAGIVLA. (b) The signal peptide prediction of Omp22: MFKRSITAAALGAAVMAFAGSAFA. (c) The signal peptide prediction of Omp28: MNTRASNFLAASFSTIMLVGAFSLPAFA.

The signal peptides of Omp22, Omp19 and Omp28 were predicted separately by the SignalP-5.0. Signal peptide sequence of the Omp19 was MGISKASLLSLAAAGIVLA (Fig. 1a); signal peptide sequence of the Omp22 was MFKRSITAAALGAAVMAFAGSAFA (Fig. 1b); signal peptide sequence of the Omp28 was MNTRASNFLAASFSTIMLVGAFSLPAFA (Fig. 1c). All of the three signal peptide sequences were removed from the epitope prediction of Omp22, Omp19 and Omp28.

### Accessible and hydrophilic region of the proteins

**Fig. 2.**
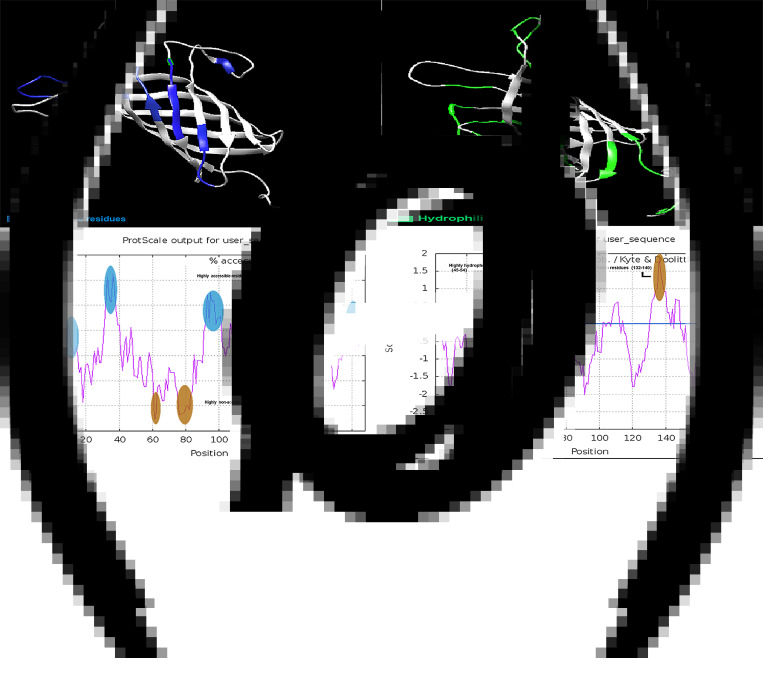
Solvent accessible and hydrophilic regions of Omp22. (a) The blue residues show the surface-accessible regions of Omp22 as tertiary structure. (b) The accessible residues are displayed as a ProtScale plot. The residues exceeding the threshold (6.0) will be considered surface accessible residues. (c) The green residues displayed the hydrophilic regions of Omp22 as tertiary structure. (d) two highly hydrophobic area (aa45–54) and (aa132–140) is marked in brown on the ProtScale hydrophobic plot.

The accessibility and hydrophilic regions of the Omp22 protein residues and their locations in three-dimensional structures are shown in ×Figure 2a and c. Amino acids 61–62 and 75–82 are considered highly unreachable residues due to a surface accessibility score below 5.0 (Fig. 2b). Amino acids 49–54 and 132–140 were estimated to be highly hydrophobic fragments (Fig. 2d). In the prediction of T/B epitopes, the residual regions of hydrophobicity and inaccessibility are neglected and they are unlikely to bind to specific antibodies.

**Fig. 3.**
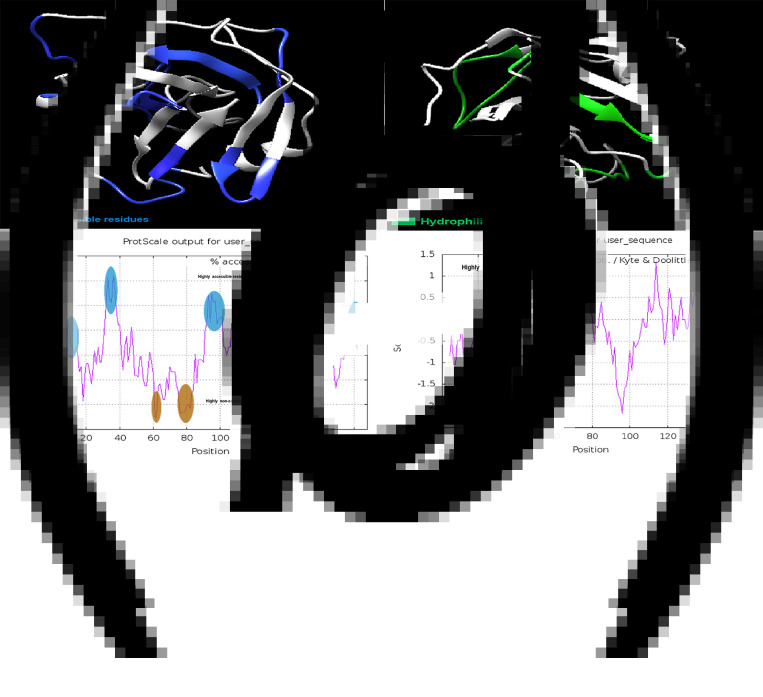
Solvent accessible and hydrophilic regions of Omp19. (a) The blue residues show the surface-accessible regions of Omp19 as tertiary structure. (b) The accessible residues are displayed as a ProtScale plot. The residues exceeding the threshold (6.0) will be considered surface accessible residues. (c) The green residues displayed the hydrophilic regions of Omp19 as tertiary structure. (d) A highly hydrophobic area (aa70–78) is marked in brown on the ProtScale hydrophobic plot.

The accessibility and hydrophobic residues of the Omp19 protein and their positions in the 3D structure are shown in Figure 3a and c. Amino acids 102–107 were considered highly inaccessible areas (Fig. 3b). The residues between amino acids 70–78 were predicted to be highly hydrophobic fragments (Fig. 3d).

**Fig. 4.**
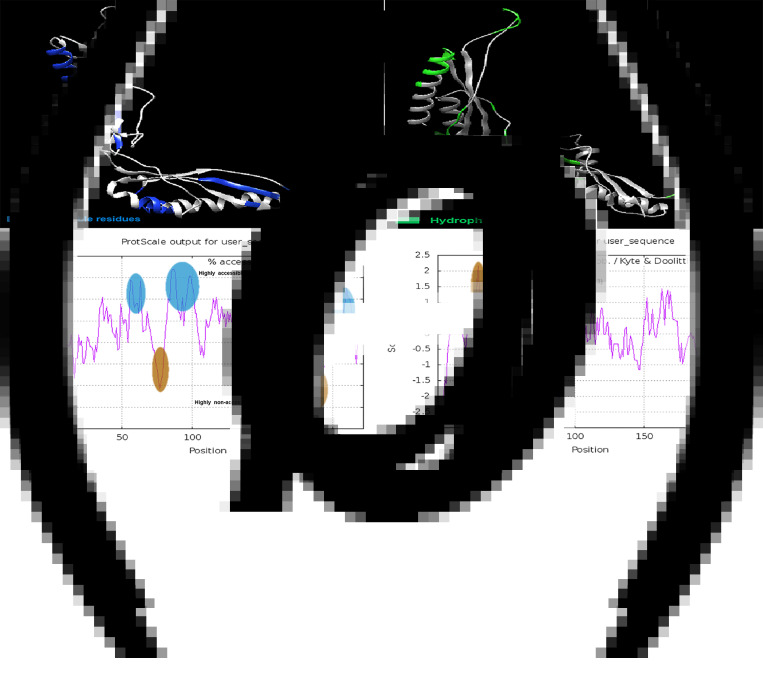
Solvent accessible and hydrophilic regions of Omp28. (a) The blue residues indicate the surface-accessible regions of Omp28 as tertiary structure. (b) The accessible residues are shown as a ProtScale plot. The residues exceeding the threshold (6.0) will be considered surface accessible residues. (c) The green residues displayed the hydrophilic regions of Omp28 as tertiary structure. (d) A highly hydrophobic area (aa27–33) is marked in brown on the ProtScale hydrophobic plot.

The accessibility and hydrophobic residues of the Omp28 protein and their positions in the 3D structure are shown in ×Figure 4a and c. Amino acids 75–79 and amino acids 179–194 were considered highly inaccessible areas (Fig. 4b). The residues between amino acids 27–33 were predicted to be highly hydrophobic fragments (Fig. 4d).

### Tertiary structure of proteins

**Fig. 5.**
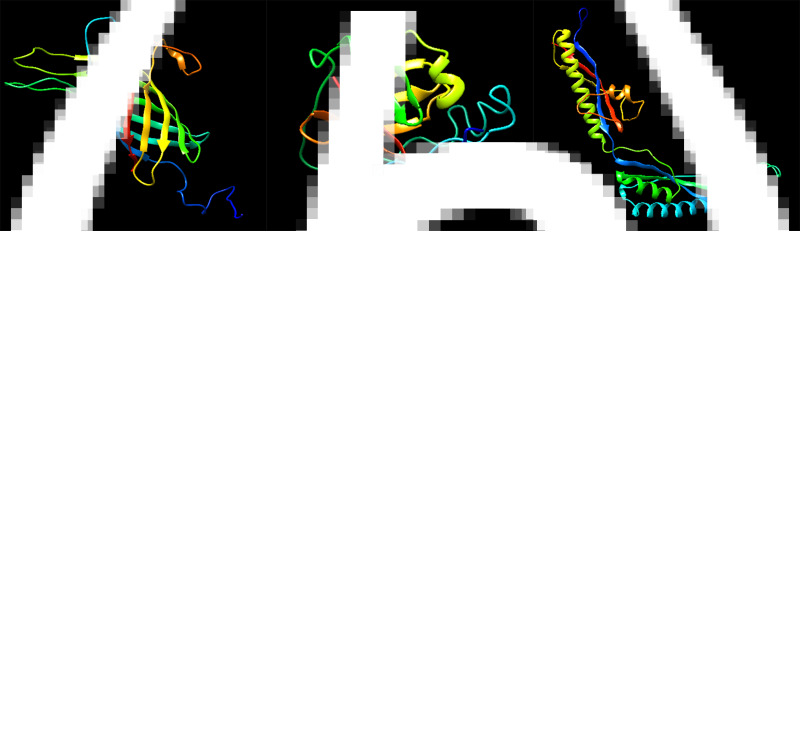
Tertiary structure of protein. Multi-coloured ribbon and coil structure represents the helix, sheets and coiled secondary structure component of the 3D model obtained for the protein. (a) Omp22. (b) Omp19. (c) Omp28.

The I-TASSER online program was employed to model the 3D structure of proteins (Fig. 5). The C score of the Omp22 prediction model was shown as −0.56, and the TM score and the RMSD of the model are shown as 0.64 ± 0.13 and 6.4 ± 3.9 Å, respectively, so it had high reliability (Fig. 5a). The prediction model of the Omp19 was not highly reliable (Fig. 5b), and its C score was shown as −3.12. The TM and RMSD were 0.36 ± 0.12 and 12.0 ± 4.4 Å, respectively. The prediction model of the Omp28 was very reliable, with a C score of 0.94. The TM and RMSD are shown as 0.84 ± 0.08 and 3.7 ± 2.5 Å, respectively (Fig. 5c).

### Prediction of B-cell epitopes

We used online software COBEpro, SVMTriP and BepiPred to screen the predicted Epitopes of B cells and displayed them in tabular form. The B cell epitopes for Omp22, Omp19 and Omp28 are shown in Tables 1–3, respectively.

**Table 1.** B cell epitopes of Omp22

	No.	Position	Sequence	Score
COBEpro	01	158–163	FNDVKS	0.826
	02	159–164	NDVKSQ	0.810
	03	174–179	NLKNHS	0.748
	04	125–131	TSASGWE	0.742
	05	40–46	SPFASRT	0.730
	06	118–123	FKAKDN	0.579
	07	116–122	TRFKAKD	0.700
	08	161–166	DVKSQV	0.723
	09	169–175	IEQRNNL	0.684
	10	123–129	NTTSASG	0.699
SVMtri	01	119–138	KAKDNTTSASGWEGGVLIGA	1.000
BepiPred	01	4–45	MGGTDYTYNDPVAAGPHDW SGNYVGAQVGGSSSKFPSPFASR	1.711–0.571
	02	85–99	TLQQSWNGNAKGKVG	1.250–0.424
	03	120–131	AKDNTTSASGWE	1.724–0.547
	04	164–174	SQVNGIEQRNN	0.659–0.399

**Table 2.** B cell epitopes of Omp19

	No.	Position	Sequence	Score
COBEpro	01	24–29	VPAGTV	0.814
	02	51–57	QSGTQVA	0.744
	03	91–98	QTKYGQGY	0.640
	04	57–62	ASLPPA	0.721
	05	34–40	LDSPTQF	0.718
	06	29–34	VQKGNL	0.764
	07	40–47	FPNAPSTD	0.699
	08	12–19	VSPPPPPA	0.695
	09	142–148	RFDGQTT	0.680
	10	125–131	YDANGGT	0.638
SVMtri	01	111–130	ANLASWAVNGKQLVLYDANG	1.000
BepiPred	01	12–53	VSPPPPPAPVNAVPAGTVQKGNL DSPTQFPNAPSTDMSAQSG	0.539–2.953
	02	57–75	ASLPPASAPDLTPGAVAGV	0.540–1.654
	03	91–106	QTKYGQGYRAGPLRCP	0.564–0.923
	04	144–153	DGQTTGGQAV	0.502–0.889

**Table 3.** B cell epitopes of Omp28

	No.	Position	Sequence	Score
COBEpro	01	64–70	EDRDLQT	0.845
	02	65–71	DRDLQT	0.839
	03	88–94	LKEPTI	0.831
	04	86–92	NNLKEPT	0.811
	05	133–138	VNDNPS	0.810
	06	7–13	TQPARIA	0.754
	07	203–209	PIAAGEN	0.731
	08	206–212	AGENSYN	0.650
	09	3–13	NQMTTQPARIA	0.605
	10	8–14	QPARIAV	0.723
SVMtri	01	41–60	REAMTANNEAMTKVLDAMKK	1.000
	02	203–222	PIAAGENSYNVSVNVVFEIK	0.845
BepiPred	01	1–12	QENQMTTQPARI	1.258–0.523
	02	39–49	AREAMTANNE	0.830–0.502
	03	62–73	GIEDRDLQTGGI	1.083–0.330
	04	80–97	VYPDDKNNLKEPTITGYS	1.512–0.565
	05	133–141	VNDNPSAVI	0.990–0.502
	06	176–185	LSRPPMPMPI	1.160–0.629
	07	197–212	APDNSVPIAAGENSYN	1.131–0.518

### Prediction of T cell epitopes

#### CD8^+^ T cell epitopes

Online software SYFPEITHI, IEDB and RANKpep were employed to analyse the CD8^+^ T cell epitope of the proteins. The analysis results of the Omp22 are shown in Tables 4–6. The analysis results of the Omp19 are shown in Tables 7–9. The analysis results of the Omp28 are shown in Tables 10–12.

**Table 4.** The CD8^+^ T cell epitopes of Omp22 by SYFPEITHI

Allele	No.	Position	Sequence	Score
HLA-A*02:01	01	48–56	ALGGIVVGK	21
	02	142–150	QALSGPLSV	21
	03	135–143	LIGAGVEQA	20
	04	126–134	SASGWEGGV	19
	05	134–142	VLIGAGVEQ	19
	06	143–151	ALSGPLSVK	19
	07	2–10	DMMGGTDYT	18
	08	61–69	GNIVFGAEL	18
	09	47–55	GALGGIVVG	17
	10	128–136	SGWEGGVLI	17
HLA-A*24:02	01	72–80	NFAEAEHRI	18
	02	41–49	PFASRTGAL	17
	03	128–136	SGWEGGVLI	14
	04	167–175	NGIEQRNNL	14
	05	10–18	TYNDPVAAG	13
	06	78–86	HRIGHGGTL	13
	07	112–120	GYGVTRFKA	13
	08	30–38	QVGGSSSKF	12
	09	34–42	SSSKFPSPF	12
	10	61–69	GNIVFGAEL	12

**Table 5.** The CD8^+^ T cell epitopes of Omp22 by IEDB

Allele	No.	Position	Sequence	Percentile rank
HLA-A*02:01	01	9–17	YTYNDPVAA	0.34
	02	128–136	SGWEGGVLI	1.9
	03	142–150	QALSGPLSV	2.4
	04	100–108	YTFDKTLVY	2.8
	05	57–65	NMQNGNIVF	2.8
	06	135–143	LIGAGVEQA	3.0
	07	3–11	MMGGTDYTY	3.6
	08	68–76	ELEGNFAEA	4.1
	09	72–80	NFAEAEHRI	4.6
	10	2–10	DMMGGTDYT	4.6
HLA-A*24:02	01	107–115	VYGTAGYGV	0.13
	02	57–65	NMQNGNIVF	0.51
	03	72–80	NFAEAEHRI	0.57
	04	99–107	GYTFDKTLV	1.1
	05	158–166	RFNDVKSQV	1.3
	06	110–118	TAGYGVTRF	1.8
	07	151–159	KAEYDFQRF	1.9
	08	180–188	IKAGLNYKF	2.0
	09	3–11	MMGGTDYTY	2.1
	10	94–102	AKGKVGYTF	2.2

**Table 6.** The CD8^+^ T cell epitopes of Omp22 by RANKPEP

Allele	No.	Position	Sequence	Score
HLA-A*02:01	01	158–168	RFNDVKSQV	55.0
	02	135–143	LIGAGVEQA	54.0
	03	174–182	NIKNHSIKA	53.0
	04	142–150	QALSGPLSV	53.0
HLA-A*24:02	01	72–80	NFAEAEHRI	12.3
	02	41–49	PFASRTGAL	12.2
	03	107–115	VYGTAGYGV	9.6
	04	14–22	PVAAGPHDW	3.9

**Table 7.** The CD8^+^ T cell epitopes of Omp19 by SYFPEITHI

Allele	No.	Position	Sequence	Score
HLA-A*02:01	01	68–76	TPGAVAGVL	25
	02	80–88	SLGGQSCKI	24
	03	128–136	ANGGTVASL	22
	04	117–125	AVNGKQLVL	21
	05	5–13	SRLGNLDNV	20
	06	148–156	TTGGQAVTL	20
	07	73–81	VAGVWNASL	19
	08	52–60	QSGTQVASL	18
	09	105–113	RCPGELANL	18
	10	110–118	LANLASWAV	18
HLA-A*24:02	01	136–144	LYSSGQGRF	20
	02	33–41	GNLDSPTQF	14
	03	102–110	GPLRCPGEL	13
	04	60–68	LPPASAPDL	12
	05	73–81	VAGVWNASL	12
	06	80–88	SLGGQSCKI	12
	07	105–113	RCPGELANL	11
	08	2–10	CQSSRLGNL	11
	09	52–60	QSGTQVASL	11
	10	94–102	KYGQGYRAG	11

**Table 8.** The CD8^+^ T cell epitopes of Omp19 by IEDB

Allele	No.	Position	Sequence	Percentile rank
HLA-A*02:01	01	124–132	VLYDANGGT	1.3
	02	71–79	GAVAGVWNA	1.7
	03	68–76	LTPGAVAGV	1.7
	04	80–88	SLGGQSCKI	2.1
	05	55–63	TQVASLPPA	2.7
	06	117–126	AVNGKQLVL	2.9
	07	146–154	GQTTGGQAV	3.9
	08	39–47	TQFPNAPST	4.0
	09	12–20	NVSPPPPPA	4.0
	10	64–72	SAPDLTPGA	4.1
HLA-A*24:02	01	136–144	LYSSGQGRF	0.1
	02	115–123	SWAVNGKQL	0.59
	03	125–133	LYDANGGTV	0.79
	04	33–41	GNLDSPTQF	2.9
	05	108–116	GELANLASW	4.0
	06	117–125	AVNGKQLVL	5.2
	07	148–156	TTGGQAVTL	5.7
	08	5–13	SRLGNLDNV	5.7
	09	68–76	LTPGAVAGV	6.8
	10	94–102	KYGQGYRAG	6.9

**Table 9.** The CD8^+^ T cell epitopes of Omp19 by RANKPEP

Allele	No.	Position	Sequence	Score
HLA-A*02:01	01	80–88	SLGGQSCKI	76.0
	02	117–125	AVNGKQLVL	63.0
	03	68–76	LTPGAVAGV	50.0
HLA-A*24:02	01	136–144	LYSSGQGRF	16.3
	02	115–123	SWAVNGKQL	16.0
	03	125–133	LYDANGGTV	8.0

**Table 10.** The CD8^+^ T cell epitopes of Omp28 by SYFPEITHI

Allele	No.	Position	Sequence	Score
HLA-A*02:01	01	101–109	SLTVRVREL	27
	02	114–122	KILDESVTL	26
	03	25–33	DMAILNLSV	23
	04	162–170	AAGVGLGRV	22
	05	194–202	LAAAPDNSV	22
	06	165–173	VGLGRVVEI	21
	07	26–34	MAILNLSVL	20
	08	72–80	GINIQPIYV	20
	09	148–156	AVANAIAKA	20
	10	157–165	KTLADAAGV	20
HLA-A*24:02	01	80–88	VYPDDKNNL	25
	02	165–173	VGLGRVVEI	16
	03	107–115	RELANVGKI	15
	04	213–221	VSVNVVFEI	15
	05	114–122	KILDESVTL	14
	06	177–185	SRPPMPMPI	14
	07	151–159	NAIAKAKTL	13
	08	196–205	AAPDNSVPI	13
	09	21–29	TASPDMAIL	12
	10	26–34	MAILNLSVL	12

**Table 11.** The CD8^+^ T cell epitopes of Omp28 by IEDB

Allele	No.	Position	Sequence	Percentile rank
HLA-A*02:01	01	114–122	KILDESVTL	0.07
	02	72–80	GINIQPIYV	0.36
	03	18–26	GMMTASPDM	0.71
	04	132–140	LVNDNPSAV	0.75
	05	157–165	KTLADAAGV	0.84
	06	131–139	NLVNDNPSA	0.84
	07	148–156	AVANAIAKA	1.2
	08	50–58	AMTKVLDAM	1.5
	09	209–217	NSYNVSVNV	2.0
	10	205–213	AAGENSYNV	2.1
HLA-A*24:02	01	80–88	VYPDDKNNL	0.13
	02	210–218	SYNVSVNVV	0.31
	03	182–190	PMPIARGQF	0.84
	04	211–219	YNVSVNVVF	1.6
	05	177–185	SRPPMPMPI	2.5
	06	114–122	KILDESVTL	2.7
	07	213–221	VSVNVVFEI	3.1
	08	165–173	VGLGRVVEI	3.2
	09	73–81	INIQPIYVY	4.1
	10	196–204	AAPDNSVPI	4.4

**Table 12.** The CD8^+^ T cell epitopes of Omp28 by RANKPEP

Allele	No.	Position	Sequence	Score
HLA-A*02:01	01	114–122	KILDESVTL	82.0
	02	101–109	SLTVRVREL	77.0
	03	148–156	AVANAIAKA	73.0
	04	4–12	QMTTQPARI	71.0
	05	50–58	AMTKVLDAM	69.0
HLA-A*24:02	01	80–88	VYPDDKNNL	8.7
	02	101–109	SLTVRVREL	6.7
	03	210–218	SYNVSVNVV	6.0
	04	108–116	ELANVGKIL	5.6
	05	4–12	QMTTQPARI	5.3

#### CD4^+^ T cell epitopes

Online software SYFPEITHI, IEDB and RANKpep were employed to analyse the CD4^+^ T cell epitope of the proteins. The analysis results of the Omp22 are shown in Tables 13–15. The analysis results of the Omp19 are shown in Tables 16–18. The analysis results of the Omp28 are shown in Tables 19–21.

**Table 13.** The CD4^+^ T cell epitopes of Omp22 by SYFPEITHI

Allele	No.	Position	Sequence	Score
HLA-DRB*07:01	01	95–109	KGKVGYTFDKTLVYG	30
	02	137–151	GAGVEQALSGPLSVK	30
	03	83–97	GGTLQQSWNGNAKGK	24
	04	97–111	KVGYTFDKTLVYGTA	24
	05	104–118	KTLVYGTAGYGVTRF	24
	06	4–18	MGGTDYTYNDPVAAG	22
	07	28–42	GAQVGGSSSKFPSPF	22
	08	38–52	FPSPFASRTGALGGI	22
	09	62–76	NIVFGAELEGNFAEA	22
	10	133–147	GVLIGAGVEQALSGP	22
HLA-DRB*09:01	–	–	–	–

**Table 14.** The CD4^+^ T cell epitopes of Omp22 by IEDB

Allele	No.	Position	Sequence	Percentile rank
HLA-DRB*07:01	01	136–150	IGAGVEQALSGPLSV	12.00
	02	137–151	GAGVEQALSGPLSVK	13.00
	03	138–152	AGVEQALSGPLSVKA	14.00
	04	103–117	DKTLVYGTAGYGVTR	15.00
	05	102–116	FDKTLVYGTAGYGVT	15.00
	06	104–118	KTLVYGTAGYGVTRF	15.00
	07	101–115	TFDKTLVYGTAGYGV	15.00
	08	105–119	TLVYGTAGYGVTRFK	15.00
	09	155–169	DFQRFNDVKSQVNGI	16.00
	10	96–110	GKVGYTFDKTLVYGT	16.00
HLA-DRB*09:01	01	138–152	AGVEQALSGPLSVKA	5.40
	02	137–151	GAGVEQALSGPLSVK	5.70
	03	136–150	IGAGVEQALSGPLSV	6.00
	04	135–149	LIGAGVEQALSGPLS	12.00
	05	84–98	GTLQQSWNGNAKGKV	13.00
	06	139–153	GVEQALSGPLSVKAE	13.00
	07	134–148	VLIGAGVEQALSGPL	13.00
	08	140–154	VEQALSGPLSVKAEY	15.00
	09	174–188	NLKNHSIKAGLNYKF	17.00
	10	86–100	LQQSWNGNAKGKVGY	20.00

**Table 15.** The CD4^+^ T cell epitopes of Omp22 by RANKPEP

Allele	No.	Position	Sequence	Score
HLA-DRB*07:01	01	159–167	FNDVKSQVN	11.1
	02	154–162	YDFQRFNDV	11.0
	03	53–61	VVGKNMQNG	9.2
	04	43–51	ASRTGALGG	8.9
HLA-DRB*09:01	01	26–34	YVGAQVGGS	16.4
	02	100–108	YTFDKTLVY	15.9
	03	90–98	WNGNAKGKV	14.8
	04	22–30	WSGNYVGAQ	14.4

**Table 16.** The CD4^+^ T cell epitopes of Omp19 by SYFPEITHI

Allele	No.	Position	Sequence	Score
HLA-DRB*07:01	01	38–52	PTQFPNAPSTDMSAQ	32
	02	107–121	PGELANLASWAVNGK	24
	03	57–71	VASLPPASAPDLTPG	22
	04	70–84	PGAVAGVWNASLGGQ	22
	05	122–136	QLVLYDANGGTVASL	22
	06	133–147	VASLYSSGQGRFDGQ	20
	07	47–61	TDMSAQSGTQVASLP	18
	08	113–127	LASWAVNGKQLVLYD	18
	09	141–155	QGRFDGQTTGGQAVT	18
	10	19–33	PAPVNAVPAGTVQKG	16
HLA-DRB*09:01	–	–	–

**Table 17.** The CD4^+^ T cell epitopes of Omp19 by IEDB

Allele	No.	Position	Sequence	Percentile rank
HLA-DRB*07:01	01	106–120	CPGELANLASWAVNG	7.20
	02	108–122	GELANLASWAVNGKQ	7.20
	03	104–118	LRCPGELANLASWAV	7.20
	04	107–121	PGELANLASWAVNGK	7.20
	05	105–119	RCPGELANLASWAVN	7.20
	06	109–123	ELANLASWAVNGKQL	16.00
	07	110–124	LANLASWAVNGKQLV	16.00
	08	111–125	ANLASWAVNGKQLVL	27.00
	09	113–127	LASWAVNGKQLVLYD	27.00
	10	112–126	NLASWAVNGKQLVLY	27.00
HLA-DRB*09:01	01	54–68	GTQVASLPPASAPDL	4.10
	02	51–65	AQSGTQVASLPPASA	4.50
	03	52–66	QSGTQVASLPPASAP	4.70
	04	53–67	SGTQVASLPPASAPD	4.70
	05	55–69	TQVASLPPASAPDLT	4.80
	06	62–76	PASAPDLTPGAVAGV	6.80
	07	63–77	ASAPDLTPGAVAGVW	6.90
	08	65–79	APDLTPGAVAGVWNA	7.10
	09	64–78	SAPDLTPGAVAGVWN	7.10
	10	66–80	PDLTPGAVAGVWNAS	7.20

**Table 18.** The CD4^+^ T cell epitopes of Omp19 by RANKPEP

Allele	No.	Position	Sequence	Score
HLA-DRB*07:01	01	98–106	GYRAGPLRC	18.3
	02	75–83	GVWNASLGG	11.4
	03	24–32	AVPAGTVQK	10.9
HLA-DRB*09:01	01	116–124	WAVNGKQLV	10.6
	02	77–85	WNASLGGQS	8.5
	03	96–104	GQGYRAGPL	5.5

**Table 19.** The CD4^+^ T cell epitopes of Omp28 by SYFPEITHI

Allele	No.	Position	Sequence	Score
HLA-DRB*07:01	01	93–107	ITGYSVSTSLTVRVR	32
	02	9–23	PARIAVTGEGMMTA	30
	03	95–109	GYSVSTSLTVRVREL	30
	04	113–127	GKILDESVTLGVNQG	30
	05	17–31	EGMMTASPDMAILNL	28
	06	109–113	LANVGKILDESVTLG	28
	07	191–205	RTMLAAAPDNSVPIA	28
	08	208–212	ENSYNVSVNVVFEIK	28
	09	15–39	TGEGMMTASPDMAIL	24
	10	130–144	LNLVNDNPSAVINEA	24
HLA-DRB*09:01	–	–	–	–

**Table 20.** The CD4^+^ T cell epitopes of Omp28 by IEDB

Allele	No.	Position	Sequence	Percentile rank
HLA-DRB*07:01	01	92–106	TITGYSVSTSLTVRV	0.96
	02	90–104	EPTITGYSVSTSLTV	1.20
	03	93–107	ITGYSVSTSLTVRVR	1.40
	04	91–105	PTITGYSVSTSLTVR	1.70
	05	94–108	TGYSVSTSLTVRVRE	1.70
	06	95–109	GYSVSTSLTVRVREL	2.10
	07	96–110	YSVSTSLTVRVRELA	3.40
	08	205–219	AAGENSYNVSVNVVF	7.50
	09	207–221	GENSYNVSVNVVFEI	7.80
	10	208–222	ENSYNVSVNVVFEIK	9.20
HLA-DRB*09:01	01	188–202	GQFRTMLAAAPDNSV	0.75
	02	90–104	EPTITGYSVSTSLTV	0.78
	03	185–199	IARGQFRTMLAAAPD	0.84
	04	92–106	TITGYSVSTSLTVRV	0.89
	05	186–200	ARGQFRTMLAAAPDN	0.90
	06	184–198	PIARGQFRTMLAAAP	0.90
	07	187–201	RGQFRTMLAAAPDNS	0.90
	08	91–105	PTITGYSVSTSLTVR	1.10
	09	93–107	ITGYSVSTSLTVRVR	1.30
	10	94–108	TGYSVSTSLTVRVRE	1.60

**Table 21.** The CD4^+^ T cell epitopes of Omp28 by RANKPEP

Allele	No.	Position	Sequence	Score
HLA-DRB*07:01	01	153–161	IAKAKTLAD	16.0
	02	82–90	PDDKNNLKE	13.3
	03	96–104	YSVSTSLTV	11.5
	04	103–111	TVRVRELAN	10.8
	05	196–204	AAPDNSVPI	8.5
HLA-DRB*09:01	01	151–159	NAIAKAKTL	15.2
	02	211–219	YNVSVNVVF	10.4
	03	37–45	AKTAREAMT	8.5
	04	96–104	YSVSTSLTV	8.1
	05	44–52	MTANNEAMT	6.1

### Overlapping epitopes

These results were compared to find selected sequences with overlapping regions that were identified as dominant B and T epitopes of proteins (Tables 22–24).

**Table 22.** The dominant linear B and T epitopes of Omp22

	Allele	Position	Sequence	Immunogenicity	Antigenicity
B epitopes	–	120–138	AKDNTTSASGWEGGVLIGA		
		164–174	SQVNGIEQRNN		
CD8 + T epitopes	HLA-A*02:01	142–150	QALSGPLSV	−0.2535	0.3431
	HLA-A*24:02	41–49	PFASRTGAL	−0.0439	−0.2589
		107–115	VYGTAGYGV	0.1398	1.4380
CD4 + T epitopes	HLA-DRB*07:01	154–167	YDFQRFNDVKSQVN		
	HLA-DRB*09:01	90–98	WNGNAKGKV

**Table 23.** The dominant linear B and T epitopes of Omp19

	Allele	Position	Sequence	Immunogenicity	Antigenicity
B epitopes	–	24–47	VPAGTVQKGNLDSPTQFPNAPSTD		
		109–130	ELANLASWAVNGKQLVLYDANG		
		142–153	RFDGQTTGGQAV		
CD8 + T epitopes	HLA-A*02:01	80–88	SLGGQSCKI	−0.3949	2.5675
		117–125	AVNGKQLVL	−0.2722	0.7816
		146–154	GQTTGGQAV	0.0416	2.4385
	HLA-A*24:02	136–144	LYSSGQGRF	−0.2659	0.2897
CD4 + T epitopes	HLA-DRB*07:01	113–126	LASWAVNGKQLVY		
	HLA-DRB*09:01	–	–

**Table 24.** The dominant linear B and T epitopes of Omp28

	Allele	Position	Sequence	Immunogenicity	Antigenicity
B epitopes	–	203–214	PIAAGENSYNVS		
		41–60	REAMTANNEAMTKVLDAMKK		
		1–12	QENQMTTQPARI		
		63–73	IEDRDLQTGGI		
CD8 + T epitopes	HLA-A*02:01	114–122	KILDESVTL	0.0180	0.0836
		148–156	AVANAIAKA	0.0765	0.1095
	HLA-A*24:02	80–88	VYPDDKNNL	−0.1664	−0.3953
		210–218	SYNVSVNVV	−0.0586	1.4632
CD4 + T epitopes	HLA-DRB*07:01	96–111	YSVSTSLTVRVRELAN		
	HLA-DRB*09:01	90–104	EPTITGYSVSTSLTV		

### Class I immunogenicity and antigenic prediction

The immunogenicity and antigenicity analysis of CD8^+^ T cell dominant epitopes were performed by the MHC I immunogenicity prediction tool of the IEDB server and VaxiJen 2.0 server, respectively. Both immunogenicity and antigenicity were positive for epitope, which could be further analysed. The results are shown in Tables 22–24.

### Design of the multi-epitope vaccine construct

To construct the final chimaeric subunit vaccine sequence, the predicted epitope of B cells was used as a template and compared with the T cell epitope. Epitopes whose sequences overlap with the B cell epitope were preferentially chosen for the final vaccine construct (Tables 25–27). Finally, six sequences were selected as constructs, including sequences 120–138, 154–174 of Omp22, and epitope sequences 24–47, 109–130, 142–153 of Omp19 and sequence 41–73 of Omp28. These epitopes were connected by amino acid linkers. HEYGAEALERAG and GGGS linkers bind the T-epitopes and the B-epitopes, while the carrier sequences connect the N-terminal and C-terminal via the EAAAK linker (Table 28).

**Table 25.** Comparative analysis of all predicted B cell, HLA-I and HLA-II epitopes of Omp22

S. no	Position	Final B Epitope （Omp22）	HLA-II	HLA-I
1	120–138	AKDNTTSASGWEGGVLIGA	–	–
2	164–174	SQVNGIEQRNN	YDFQRFNDVKSQVN	
3	107–115	–	–	VYGTAGYGV
4	90–98	–	WNGNAKGKV	–

**Table 26.** Comparative analysis of all predicted B cell, HLA-I and HLA-II epitopes of Omp19

S. no.	Position	Final B epitope （Omp19）	HLA-II	HLA-I
1	24–47	VPAGTVQKGNLDSPTQFPNAPSTD		
2	109–130	ELANLASWAVNGKQLVLYDANG	LASWAVNGKQLVY	–
3	142–153	RFDGQTTGGQAV	–	GQTTGGQAV

**Table 27.** Comparative analysis of all predicted B cell, HLA-I and HLA-II epitopes of Omp28

S. no.	Position	Final B epitope （Omp28）	HLA-II	HLA-I
1	203–214	PIAAGENSYNVS		
2	41–73	REAMTANNEAMTKVLDAMKKAGIEDRDLQTGGI		
3	1–12	QENQMTTQPARI		
4	96–111	–	YSVSTSLTVRVRELAN	–
5	90–104	–	EPTITGYSVSTSLTV	–

**Table 28.** Predict allergenicity, antigenicity and solubility of vaccine structure

Epitope sequence position with carrier	The complete sequence of Vaccine construct	Allergenicity (threshold 35%)	Antigenicity (threshold 0.5)	Solubility (threshold 0.5)
Omp22(120–138, 154–174) and Omp19 (24–47, 109–130, 142–153) and Omp28 (41–73) epitope with carrier sequence	**EAAAK**MAENPNIDDLPAPLLAALGAADLALATVNDLIANLRERAEETRAETRTRVEERRARLTKFQEDLPEQFIELRDKFTTEELRKAAEGYLEAATNRYNELVERGEAALQRLRSQTAFEDASARAEGYVDQAVELTQEALGTVASQTRAVGERAAKLVGIEL**EAAAKAKFVAAWTLKAAAGGGS**AKDNTTSASGWEGGVLIGA**GGGS**YDFQRFNDVKSQVNGIEQRNN**GGGSAKFVAAWTLKAAAGGGS**VPAGTVQKGNLDSPTQFPNAPSTD**HEYGAEALERAG**ELANLASWAVNGKQLVLYDANG**HEYGAEALERAG**RFDGQTTGGQAV**HEYGAEALERAG**REAMTANNEAMTKVLDAMKKAGIEDRDLQTGGI**HEYGAEALERAGAKFVAAWTLKAAAGGGS**	7.37–11.30	0.788	0.866

### Allergenicity, antigenicity and solubility evaluation

The online software SDAP was employed to predict the allergenicity of the vaccine construct. The similarity (%) between the sequence of the construct and the most similar template was between 7.37 and 11.30, which was lower than the threshold of 35%, so the vaccine construct was considered non-allergenic. The antigenicity of this vaccine sequence was analysed using VaxiJen 2.0 service software. The antigen value of 0.788 was predicted, which was higher than the threshold value of 0.5, so the vaccine construct was considered to have good antigenicity. The vaccine construct was soluble with SOLpro SVM value 0.866, which was considered to have good solubility (Table 28).

### Prediction of the secondary structure of the vaccine construct

**Fig. 6.**
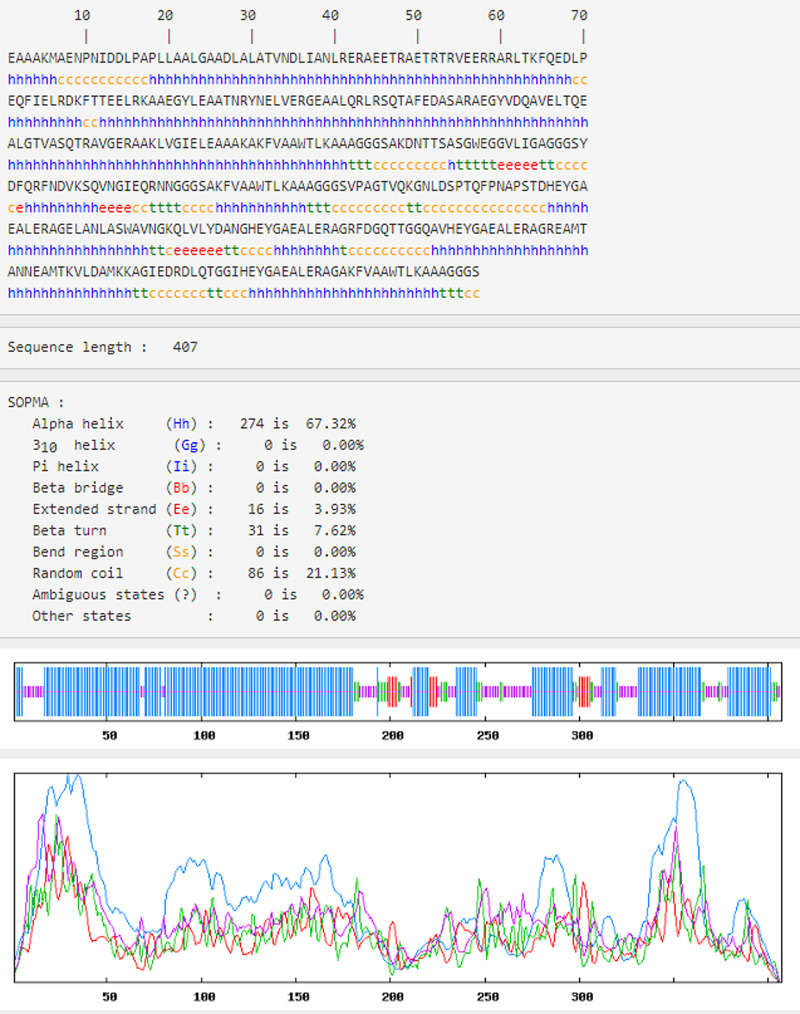
Analysis of the secondary structure of the vaccine construct by SOMPA. The sequence length of the vaccine construct is 407 amino acids. The blue h is *Alpha helix* and accounts for 67.32%, the red e is *extended strand* and accounts for 7.62%, the yellow c is *random coil* and accounts for 21.13%, the green t is *Beta turn* and accounts for 3.93%.

Using SOPMA server to analyse the secondary structure of the vaccine. The results have shown that the structure had 67.32% *alpha-helix*, 3.39% *extended strand*, 7.62% *Beta turn* and 21.13% *random coil* (Fig. 6).

### Physicochemical analysis of the vaccine construct

The vaccine construct was made up of 407 amino acids and had a molecular weight of approximately 43 kDa. The molecular formula was C_1855_H_2947_N_551_O_612_S_4_. The theoretical pI value was 4.95 and contained 45 strongly alkaline ( + ) amino acids (K, R) and 61 strongly acidic (–) amino acids (D, E). The instability index was 29.87 (the instability index of the stable protein <40), which was predicted to be a stable protein. The grand average of hydropathicity (GRAVY): −0.439 (GRAVY ranges from −2 to 2, negative values indicate hydrophilic proteins) and were classified as a hydrophilic construct. The results show that the vaccine construct has good characteristics of initiating an immunogenic response.

### Prediction and verification of the 3D structure of the vaccine construct

**Fig. 7.**
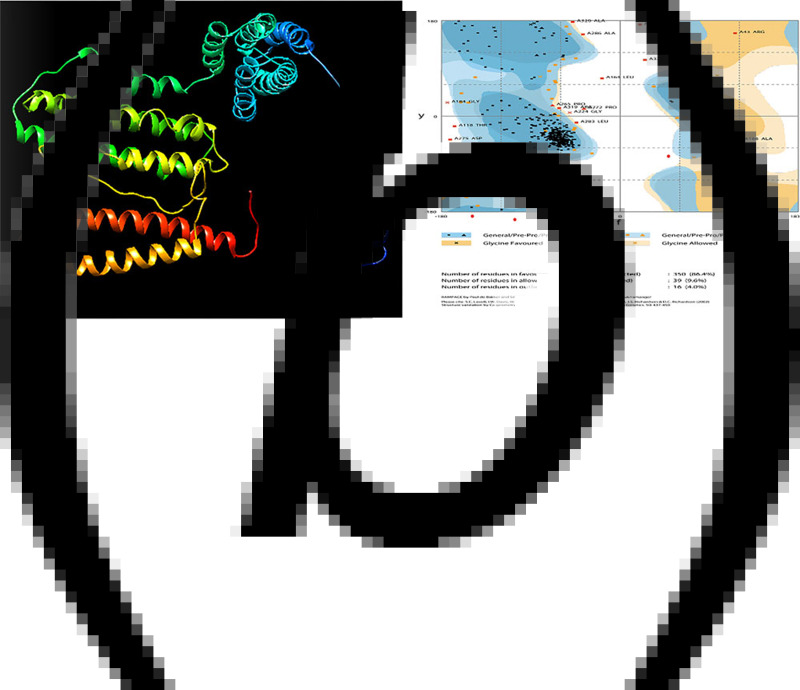
The 3D structure prediction and validation of the vaccine construct. (a) The 3D structure of model construct. (b) Ramachandran diagram of the mock vaccine, showing 96.0 residues in the allowable range. Ramachandran plot takes the angles of *Phi* and *Pis* as the abscissa and ordinate. *Phi* is the rotation angle of C−N bond on the left side of *α* carbon in a peptide unit, and *Pis* is the rotation angle of C−C bond on the right side of *α* carbon. The area inside the yellow coil is completely allowed, the area inside the blue coil is allowed and the area outside the blue coil is not allowed. When the scatter in the blue coil and the yellow coil exceeds 90%, the tertiary structure of the model conforms to rules of stereochemistry.

The results showed that the C-score of the three-dimensional (3D) model was shown as −1.52, and the TM score and RMSD of the model were 0.53 ± 0.15 and 10.4 ± 4.6 Å, respectively (Fig. 7a). The structure validation was achieved by Ramachandran graph analysis. The results showed that there were 86.4%, 9.6% and 4.0% residues in favourable, allowable and outlier regions, respectively (Fig. 7b).

## Discussion

Brucellosis is a serious infectious disease with low cure rate and complex clinical symptoms. At present, vaccine is considered to be the most effective measure to prevent Brucellosis [32]. In this study, we used bioinformatics methods to design a multi-epitope vaccine construct, which provided detailed insights into the initial stages of vaccine development.

Based on previous studies, the Omp22, Omp19 and Omp28 were evaluated as having good immunogenicity and inducing immune protection *in vivo.* Moreover, Omp22 and Omp28 were highly conservative, stable and not easily degraded, which provides favourable conditions for later vaccine construction. Therefore, it is of great significance to select the Omp22, Omp19 and Omp28 as candidate proteins for constructing a multi-epitope vaccine against *Brucella*. As far as we know, there is no multi-epitope vaccine design based on these three candidate proteins.

SignalP-5.0 server was employed for predicting the signal peptide of the Omp22, Omp19 and Omp28. We respectively removed the signal peptide sequences of Omp22, Omp19 and Omp28, namely 1–24 aa (MFKRSITAAALGAAVMAFAGSAFA), 1–19 aa (MGISKASLLSLAAAGIVLA) and 1–28 aa (MNTRASNFLAASFSTIMLVGAFSLPAFA). Signal peptides were usually located at the start of the protein translation region, affecting protein expression. To predict T/B epitopes more accurately, it was necessary to remove the signal peptide. It was found that the highly hydrophilic region of the antigen was conducive to the interaction with the antibody binding sites, and the more accessible residues on the surface of the antigen, the more conducive to the binding of the antibody [33]. Therefore, we screened out highly hydrophobic and inaccessible regions of these outer membrane proteins to ensure that the epitopes were located on the more hydrophilic and accessible residues (Figs 1 and 2).

The key to preparing an epitope vaccine is to obtain the epitopes of relative antigen [34]. Therefore, in this study, the T cell and B cell epitopes of three candidate proteins were screened by the variety of epitope prediction software, which improved the accuracy of epitope prediction [35], then dominant epitopes with both T and B cell were elected. The immune response of *Brucella* mainly depends on active T cells. However, many vaccines can only induce B cell immunity [36]. CD8^+^ T cells can effectively lyse and kill infected cells, thus exposing *Brucella* to the outside of cells and triggering other germicidal mechanisms. Therefore, we analysed the antigenicity and immunogenicity of CD8^+^ T cell epitopes to ensure that the epitope vaccine can effectively activate CD8^+^ T cells. Considering that CD4 ^+^ T cells are needed to induce appropriate antibody immune response, we also included CD4 ^+^ T cell epitopes in the vaccine construct. Our multi-epitope vaccine was designed based on T/B cell epitopes, it is possible to selectively activate specific B cells, CTL and T helper cells to achieve a fully protected and sustained immune response. Finally, six dominant epitopes were identified by a series of comparisons. There were two dominant epitopes (120–138, 154–174) from the Omp22, three dominant epitopes (24–47, 109–130, 142–153) from the Omp19 and one dominant epitope (41–73) from the Omp28.

As shown in Table 28, our vaccine construct is composed of HBHA，PADRE (as a carrier) located at the N- and C-terminal end of the vaccine sequence，six sets of T/ B cell epitopes in the middle of the vaccine construct, which were connected to each other by appropriate linkers. Using GSSS and HEYGAEALERAG cleavable linkers to separate these three domains from each other to enhance the expression of epitopes. These linkers have two key roles in the structure of epitope vaccines: firstly, to prevent the generation of binding epitopes (new epitopes) for the designed epitope vaccine; secondly, to promote immune processing and presentation of HLA-II binding epitopes [37]. Besides, for linker sequences, we usually use glycine (G) and serine (S) as component amino acids of linker sequences [38]. Because glycine (G) and serine (S) are the smallest of all amino acids and the most flexible，have no chiral carbon and can be placed between epitope sequences without affecting the conformation and function of either sides. Moreover, due to the functional characteristics of HBHA, the EAAAK linker was used to connect HBHA to the N terminal of vaccine construct as a carrier to ensure the interaction between HBHA and other vaccine fragments was minimised and provides better separation [39]. PADRE sequence has been reported to reduce the effect of human HLA-DR polymorphism and can enhance the long-term immune response by inducing CD4^+^ T cells [40]. Therefore, we added the PADRE sequence to the vaccine construct.

Finally, we constructed a multi-epitope vaccine with a length of 407 amino acids. The physicochemical, structural and immunological properties of the vaccine construct were predicted by various bioinformatics methods. It is necessary to examine any possible allergenicity at the early stage of vaccine design [41]. Our vaccine construct was shown to be non-allergenic on SDAP software, making it more effective as a candidate vaccine. The multi-epitope vaccine construct showed higher scores of antigenicity on the VaxiJen 2.0 server. The vaccine construct showed the solubility of more than 0.5 (0.886), which exhibited that the vaccine construct will be highly soluble during its heterologous expression in *E. coli*. The solubility of recombinant protein in *E. coli* is the key to many biochemical and functional studies. In short, the vaccine construct is soluble, non-allergic and antigenic peptides.

The molecular weight (MW) of the final protein is estimated to be 43 kDa. The estimated theoretical pI was 4.95, indicating that the vaccine construct was acidic. The predicted value of the instability index was 29.87, which indicated that the protein was very stable after expression, thus further confirmed its possibility. The predicted score of the GRAVY was −0.439，which shows that the protein would be a hydrophilic construct. The results show that the vaccine construct has good characteristics of initiating an immunogenic response. Analyses of the secondary structure showed that the protein mainly contained 67.32% *alpha helices*, with 7.62% *extended strand*, and they have been identified as important ‘structural antigens’ types. The 3D structure of the vaccine construct was modelled by I-TASSER. RMSD and TM scores are index to evaluate the reliability and accuracy of the prediction model. A TM-score more than 0.5 always shows the correct topology model, and C-score was used to show its confidence. Expected TM score of 0.53 ± 0.15 validated the accuracy of the model. The chimaeric structure displayed appropriate characteristics based on the Ramachandran plot's results. Ramachandran plot analysis indicates that 96% of the residues are initiated in the favoured and allowed regions, with fewer (4%) residues in the outlier region. This indicated that the quality of the whole model is acceptable. In this study, a multi-epitope vaccine construct was designed against brucellosis by immunodominant epitopes from antigens of *Brucella*, including Omp22, Omp19 and Omp28 using the combination of online bioinformatics servers. However, there is a lack of confirmation of the protective efficacy of the vaccine construct in animal models. More studies with both in vivo and in vitro methods would be designed in the future to assess the potency of the vaccine construct.

In conclusion, this study uses a large number of immunoinformatic approaches to find the vaccine construct to fight against *Brucella* infection, which provides a theoretical basis for future laboratory experiments.

## Data Availability

The authors confirm that the data supporting the findings of this study are available within the article and its references.
